# Serum Vitamin B12 Deficiency in Chronic Hemodialysis Patients

**DOI:** 10.7759/cureus.58751

**Published:** 2024-04-22

**Authors:** Mehdi Mushtaq, Muhammad Rehan Usmani, Najia Hameed, Adnan Anwar, Atif A Hashmi

**Affiliations:** 1 Nephrology, Patel Hospital, Karachi, PAK; 2 Nephrology, Lifeline Hospital, Karachi, PAK; 3 Nephrology, Sindh Institute of Urology and Transplantation, Karachi, PAK; 4 Physiology, Hamdard College of Medicine & Dentistry, Karachi, PAK; 5 Internal Medicine, Essa General Hospital, Karachi, PAK; 6 Pathology, Liaquat National Hospital and Medical College, Karachi, PAK

**Keywords:** hemodialysis, duration of hemodialysis, serum vitamin b12, chronic renal failure, vitamin b12 deficiency

## Abstract

Introduction

Essential vitamins like folate and vitamin B12 are crucial for many physiological functions. Patients with renal failure undergoing regular hemodialysis in the general population may experience harmful effects from vitamin B12 deficits. Therefore, this study aimed to determine the frequency of vitamin B12 deficiency in hemodialysis patients and its association with other clinical parameters.

Methods

This cross-sectional study was conducted at the dialysis unit of Patel Hospital and Lifeline Hospital, Karachi, using a non-probability consecutive sampling technique after obtaining ethical approval from Lifeline Hospital (LLH/HR/02-22). The study duration was six months, from January 10, 2023, to July 22, 2023. A total of 135 adult renal failure patients with ages >18 and <70 years on maintenance hemodialysis for >1 year were included in the study. The chi-square test was used to determine the association between vitamin B12 deficiency and age and gender. A p-value of 0.05 was considered statistically significant.

Results

The study findings showed that out of 135 patients, 82 (60.7%) were males and 53 (39.3%) were females, with a mean age of 50.80 ± 10.03 years. The duration of hemodialysis was approximately 1-2 years in 98 (72.6%) patients, 2-3 years in 27 (20.0%) patients, and 3-4 years in only 9 (6.7%) patients. The mean serum vitamin B-12 levels were 411.61 ± 224.95 pg/ml, with 30 (22.2%) of the subjects being deficient. In terms of duration of hemodialysis, there was a significant association (p= 0.013). Between patients with normal 4 (4%) and deficient 5 (17%) vitamin B12 and 3-4 years of hemodialysis.

Conclusion

In this study, we found that a significant proportion of patients on chronic hemodialysis had vitamin B12 deficiency. Moreover, vitamin B12 deficiency was significantly associated with duration of hemodialysis. Therefore, we recommend periodic vitamin B12 testing in hemodialysis patients to avoid any associated complications.

## Introduction

Vitamin B12, or cobalamin, is a vital nutrient essential for human well-being and largely serves as a coenzyme in one-carbon metabolism [1]. Among its additional functions, B12 helps produce blood cells and DNA, regenerate the amino acid methionine, and maintain myelin integrity, which covers nerve cells [2]. B12 is absorbed as animal products when sufficient amounts of hydrochloric acid and the glycoprotein intrinsic factor, which is expelled from the stomach's parietal cells, are secreted. Because the vitamin is retained in the tissues and can be partly reabsorbed by the gut following secretion with bile, a small amount will be sufficient to maintain an appropriate level once it has been absorbed [3]. Approximately 2-5 mg of vitamin B12 is present in the body, 0.1% of which is present in serum. The body consumes 2-5 mcg of vitamin B12 daily; therefore, it takes several years of insufficiency to deplete body reserves [4]. For adult males and females, the daily requirement for vitamin B12 is 2.4 mcg. Prevention of megaloblastic anemia and daily use facts are the basis for this suggestion [4].

Diet plays a significant role in determining vitamin B12 levels. Consumption of dietary vitamin B12 increases from 0.4 mcg/day in vegans to 7.2 mcg/day in meat eaters [5]. These eating habits are directly related to serum B12 levels. According to recent studies, supplements, fortified foods, and animal sources affect serum B12 levels equally [5]. Another crucial factor is malabsorption. Stomach inflammation and atrophy increase with age. The ensuing hypochlorhydria limits the ileal absorption of vitamin B12 by preventing its release from salivary haptocorrin. This is supported by the observation of elevated gastrin levels with aging [6]. Additional causes of malabsorption include proton pump inhibition, immunologic status, intrinsic factor status, transcobalamin polymorphisms, post-gastrectomy, bowel illness, or constipation [7].

Patients with end-stage renal disease (ESRD) have an increased risk of developing nutritional deficiencies due to drug interactions, dietary limitations, and malnutrition. In addition, the dialysis process itself may result in vitamin B deficiency, particularly in the case of folate, because of its small molecular size, which allows for clearance during hemodialysis [8]. A deficiency can appear within a few weeks because folate is not well stored in the body [9]. According to earlier studies, serum folate levels in hemodialysis patients are lower than those in the general population [9]. Serum B12 levels in hemodialysis patients have been observed to be comparable to or above those in the typical range because of the greater molecular size of B12 and the difficulties in eliminating it during hemodialysis [9].

B12 deficiency is common in hemodialysis patients [10]. Patients on dialysis typically consume inadequate amounts of food, which puts them at risk of B12 insufficiency [11]. Additionally, high levels of electrolytes found in food sources of vitamin B12 make them unsafe for dialysis patients, compelling them to consume only meals low in vitamin B12. Moreover, B12 deficiency in hemodialysis patients is caused by the fact that B12 is a typical middle-sized molecule that is well eliminated by contemporary high-flux dialyzers [12].

B12 deficiency is difficult to diagnose in dialysis patients. Homocysteine, methylmalonic acid (MMA), and B12 levels have not been investigated as indicators of B12 deficiency in the general population [13]. Although studies have demonstrated that chronic kidney disease (CKD), ESRD, and uremia are associated with higher blood MMA levels, it is unclear whether this is due to B12 deficiency or a separate consequence of uremia. MMA levels are higher in patients with impaired renal function, although it is assumed that this is not the cause of their levels exceeding 800 nmol/L [14]. In addition to low serum B12 levels, elevated homocysteine levels have also been observed in hemodialysis patients, with a subsequent decrease after B12 medication; however, these parameters have not been proven as diagnostic markers in this population. In addition, cases of erythropoietin resistance caused by a vitamin B12 shortage cured by supplementation have been reported [10].

Hence, it is yet unknown whether baseline serum levels of folate and vitamin B12 affect the incidence of hemodialysis, particularly in Pakistan, where dietary fortification differs from that in other nations. Therefore, this study aimed to determine the frequency of vitamin B12 deficiency in hemodialysis patients and its association with various clinical parameters.

## Materials and methods

Patients and methods

It was a prospective-cross-sectional study conducted at the dialysis unit of Patel Hospital and Lifeline Hospital, Karachi, using a non-probability consecutive sampling technique. Ethical approval of the study was obtained from Lifeline Hospital (Approval number: LLH/HR/02-22). The sample size was calculated using open epi online software for sample size calculation. Keeping the prevalence of CKD requiring hemodialysis mortality rate as reported in a study at 9.6%, the sample size was 134 at a 95% confidence level [15].

The study duration was six months, from January 10, 2023, to July 22, 2023. A total of 135 adult renal failure patients with ages >18 and <70 years of age on maintenance hemodialysis for >1 year were included in the study. Patients with renal failure on peritoneal dialysis, patients not on maintenance dialysis, patients with less than one year on dialysis, a history of a strict vegetarian diet, known cases of malabsorption syndromes, and those with a history of intestinal surgeries were excluded from the study.

After obtaining ethical approval, patients who met the inclusion and exclusion criteria were asked for their informed consent after being fully informed of the study's goals and methods and guaranteed confidentiality. A proforma created for the study was used for data collection. Demographic details were obtained from the patients, including age, gender, duration of dialysis, residential status, and frequency of hemodialysis. 

Serum vitamin B12 estimation

After informed consent, 3 ml of blood was drawn using an aseptic approach in a serum/plasma separator tube. The collected samples were delivered to the laboratory at Ziauddin University Hospital for serum vitamin B12 estimation. The electrochemiluminescence immunoassay (ECLIA) was utilized to determine vitamin B12 levels using a cobas e immunoassay analyzer. The results were reported in picograms per milliliter (pg/ml). A < 200 pg/ml level was taken as vitamin B12 deficiency. 

Statistical analysis

Data were entered and analyzed using SPSS Statistics version 26.0 (IBM Corp. Released 2019. IBM SPSS Statistics for Windows, Version 26.0. Armonk, NY: IBM Corp). Descriptive statistics such as age, hemodialysis duration, and frequency were expressed as means and standard deviations. Qualitative variables like gender, residential status, and vitamin B12 level were presented as frequencies and percentages. The chi-square test was used to determine the association between vitamin B12 deficiency and age and gender. A P value of 0.05 was considered statistically significant.

## Results

A total of 135 patients with renal failure on hemodialysis were included in the study. Of them, 82 (60.7%) were males and 53 (39.3%) were females, with a mean age of 50.80 ± 10.03 years. Most of the patients, 126 (93.3%) lived in urban areas. Most patients, 111 (82.2%) had three hemodialysis sessions per week, and 23 (17.1%) had two weekly hemodialysis sessions. The duration of hemodialysis was approximately 1-2 years in 98 (72.6%) patients, 2-3 years in 27 (20.0%) patients, and 3-4 years in only 9 (6.7%) patients. The mean serum vitamin B-12 levels were 411.61 ± 224.95 pg/ml, with 30 (22.2%) of the subjects being deficient (Table 1).

**Table 1:** General characteristics of hemodialysis patients (n=135) The data has been presented as mean ± SD and n, %. SD: standard deviation

Variables	n	%
Age (years), mean±SD	50.80	10.03
Gender		
Male	82	60.7
Female	53	39.3
Residential status		
Urban	126	93.3
Rural	9	6.7
Frequency of hemodialysis (per week)		
Once	1	0.7
Twice	23	17.1
Thrice	111	82.2
Duration of hemodialysis		
1 – 2 years	98	72.6
2 – 3 years	27	20.0
3 – 4 years	9	6.7
≥ 4 years	1	0.7
Serum vitamin B12 (pg/ml)	411.61	224.95
Deficient	30	22.2
Normal	105	77.8

The association between vitamin B12 and chronic hemodialysis concerning gender revealed that only 20 (24.4%) males and 10 (18.9%) females had vitamin B12 deficiency, although there was an insignificant difference among them (p = 0.451). Most males (n = 66, 80.5%) and 45 (84.9%) females underwent chronic hemodialysis, with an insignificant difference among them (p= 0.512) (Table 2).

**Table 2:** Gender differences in frequency of chronic hemodialysis and vitamin B12 deficiency p-value of < 0.05 was considered significant. The data has been presented as n, %.

Variables	Male	Female	Overall	p-value
	(n = 82)	(n = 53)	(n = 135)	
Vitamin B12 deficiency				
Yes, n (%)	20 (24.4%)	10 (18.9%)	30 (22.2%)	0.451
No, n (%)	62 (75.6%)	43 (81.1%)	105 (77.8%)	
Chronic hemodialysis				
Yes, n (%)	66 (80.5%)	45 (84.9%)	111 (82.2%)	0.512
No, n (%)	16 (19.5%)	8 (15.1%)	24 (17.8%)	

The association between vitamin B12 deficiency, age groups, and duration of hemodialysis revealed that 18 (60%) patients aged >50 years had vitamin B12 deficiency, and 63 (60%) patients had normal vitamin B12. Approximately 9 (30%) patients between the ages of 31 and 50 years had vitamin B12 deficiency, and 39 (37%) patients had normal vitamin B12. Approximately 3 (10%) patients in ≤ 30 years had vitamin B12 deficiency, and 3 (3%) patients had normal vitamin B12; however, there was a statistically insignificant difference among them (p>0.05). In terms of the duration of hemodialysis, there was a significant association observed between patients with normal 4 (4%) and deficient 5 (17%) vitamin B12 and 3-4 years of hemodialysis (p = 0.013). Furthermore, a statistically insignificant difference was seen in 3 and >4 years of hemodialysis between the normal and deficient levels of vitamin B12 (p > 0.05) (Table 3).

**Table 3:** Association between vitamin B12 deficiency and age groups and duration of hemodialysis. *p-value significant as <0.05. The data has been presented as n, %.

Variables		Vitamin B12 deficiency		p-value
		Normal	Deficient	
Age groups	≤ 30 years, n (%)	3 (3%)	3 (10%)	0.095
	31 – 50 years, n (%)	39 (37%)	9 (30%)	0.471
	> 50 years, n (%)	63 (60%)	18 (60%)	>0.999
Duration of hemodialysis	1-2 years, n (%)	79 (75%)	19 (63%)	0.197
	2-3 years, n (%)	21 (20%)	6 (20%)	>0.999
	3-4 years, n (%)	4 (4%)	5 (17%)	0.013*
	≥4 years, n (%)	1 (1%)	0 (0%)	0.589

The association between vitamin B12 deficiency and chronic hemodialysis revealed that approximately 27 (90.0%) hemodialysis patients were vitamin B12 deficient, and 3 (10.0%) patients without hemodialysis were vitamin B-12 deficient, with an insignificant difference among them (p = 0.206). The odds ratio indicated that subjects on chronic hemodialysis had 2.25 times more chances of being vitamin B12 deficient than those without chronic hemodialysis (Table 4).

**Table 4:** The association between vitamin B12 deficiency and chronic hemodialysis p-value of < 0.05 was considered significant. The data has been presented as n, %

Variables		Vitamin B12 deficiency		p-value	Odds ratio
		Yes	No		
Chronic hemodialysis	Yes, n (%)	27 (90.0%)	84 (80.0%)	0.206	2.250
	No, n (%)	3 (10.0%)	21 (20.0%)		

## Discussion

In this study, we evaluated vitamin B12 deficiency in hemodialysis patients and noted that over 20% of patients on chronic hemodialysis owing to chronic renal failure had vitamin B12 deficiency. Hemodialysis patients are more likely to consume insufficient folate because of their diminished appetite and restricted diets. Additionally, a significant number of hemodialysis patients take many drugs and have a high load of comorbidities, some of which may prevent them from absorbing minerals and vitamins like folate and vitamin B12 [16]. In contrast, hemodialysis patients often have serum B12 concentrations comparable to or higher than the general population [17]. Although hemodialysis patients may consume less B12 through food or supplements, the body's ability to retain substantial levels of B12 can prevent B12 insufficiency for years [16]. B12, unlike folate, is a huge intermediate molecule that is difficult to remove during hemodialysis [16]. Therefore, this study demonstrated normal and deficient levels of vitamin B12 in renal failure patients undergoing hemodialysis.

A descriptive, cross-sectional study conducted in Iran evaluated 202 hemodialysis patients. The patients were 59.6 ± 14.45 years old on average. Of them, 135 (66.8%) were men and 67 (33.2%) were women. Numerous variables impact hemodialysis effectiveness. Patient age was a factor that influenced the degree to which hemodialysis was performed. These findings demonstrated an inverse connection between hemodialysis adequacy and age, with older patients having less adequate hemodialysis [18]. Similarly, the findings of another researcher, Anees et al. [19], revealed that the competency of hemodialysis decreased with the increasing age of the patients, which had a negative effect on their QOL. Nonetheless, the outcomes of some studies, for instance, Shariati et al. [20], presented an insignificant association between hemodialysis adequacy and age. It appears that variations in the sampling technique and the studied sample size were the reasons for this discrepancy. The present study was partially consistent with the above-reported studies and indicated that most of the patients who underwent hemodialysis were males, 66 (80.5%), as compared to females, 45 (84.9%), with a mean age of 50.80 ± 10.03 years. Moreover, most of the patients were > 50 years old and underwent hemodialysis.

Another study regarding the duration of hemodialysis revealed a substantial direct relationship between the duration of hemodialysis and hemodialysis sufficiency. Longer hemodialysis sessions result in more satisfactory hemodialysis adequacy for patients [18]. Nevertheless, Roozitalab et al. [21] demonstrated that the quality of hemodialysis degraded over time and did not discover a substantial association. These findings do not agree with those of another study. It was noted that the treatment course varied widely from 3 months to 14 years. Patients seem to have adapted more to the hemodialysis process and have improved hemodialysis adequacy with extended hemodialysis treatment durations [18]. This study did not support the abovementioned research and revealed that the duration of hemodialysis ranged from 1 year to ≥ 4 years.

Another study demonstrated the gender difference in the effectiveness of hemodialysis; it was observed that women had more effective hemodialysis than men [18]. Several investigations, including the one by Hojjat [22], have demonstrated this phenomenon, although Dehvan et al.'s findings revealed no connection between gender and adequate hemodialysis [23]. This discrepancy resulted from the different gender distributions in the different studies. Muscular mass, lower physical activity, and greater nutritional regimen compliance in women contribute to more adequate hemodialysis [24]. This study had an insignificant relationship between hemodialysis and both genders (p = 0.451). Most patients who underwent hemodialysis were males, 82 (60.7%).

Patients with ESRD are more likely to experience dietary deficiencies, which can lead to vitamin B12 deficits with harmful hematologic effects. Among patients with ESRD receiving hemodialysis, a case-control study examined the effect of intramuscular B12 on renal anemia. It was revealed that the average time they had been suffering from hemodialysis was around seven years [25], which was comparable to the study by Heinz et al. [26] that revealed a baseline median serum vitamin B12 level of 350 g/mL in 650 dialysis patients. According to a prior study by Rees and Shaw, this finding indicates an increased likelihood of vitamin B12 insufficiency in dialysis patients [27]. The present study, comparable to the previously mentioned studies, found an insignificant association (p = 0.206) between vitamin B12 deficiency and hemodialysis in 30 (22.2%) patients. The mean serum vitamin B12 concentration was 411.61 ± 224.95 pg/ml.

In contrast to Stabler's study [28], Patil et al. [29] identified a high percentage (56%) of CKD patients who were vitamin B12 deficient. Because of pharmaceutical interactions, restricted eating habits, and inadequate nutrition, which could lead to vitamin B12 deficiency, patients with ESRD are more likely to experience nutritional deficiencies. These findings corroborated the present study findings and indicated that hemodialysis patients were more likely to have vitamin B12 deficiencies due to drug interactions and malnutrition.

The prevalence of CKD is rising, and it is a significant global cause of mortality and morbidity. Because of medication interactions and dietary limitations, patients with CKD are more likely to experience nutritional deficiencies. The dialysis process may result in vitamin B12 and folic acid depletion and a resulting shortage [30]. Another study by Dandge et al. [30] studied 80 cases of CKD patients ranging in age from 18 to 80 years. It was observed that 47 cases of CKD had vitamin B12 deficiency, whereas 33 cases did not. A large number of patients were older than middle age. Approximately 61.2% of patients between the ages of 40 and 60 were men, which may be because men are more likely than women to have hypertension and diabetes (risk factors for chronic kidney disease). Nearly 77.5% of the instances come from patients who live in urban regions, and 22.5% come from patients who live in rural areas. Patients in the vitamin B12 deficient group had a mean CKD duration of 6.59 ± 2.894, and those in the vitamin B12 normal group had a mean CKD duration of 5.181 ± 2.324, with a statistically significant correlation between them (p = 0.02). According to this research, the prevalence of vitamin B12 deficiency increases as CKD duration increases. The present study was inconsistent with the above-cited study and indicated that of the 135 patients undergoing hemodialysis, 82 (60.7%) were males and 53 (39.3%) were females, with a mean age of 50.80 ± 10.03 years. Most of the patients, 126 (93.3%) lived in urban areas. Most patients, 111 (82.2%), had three hemodialysis sessions per week. The mean serum vitamin B12 levels were 411.61 ± 224.95 pg/ml, with 30 (22.2%) of the patients being deficient, whereas there was an insignificant difference observed concerning chronic hemodialysis (p = 0.206).

This study had a few limitations. Although it was a prospective study, long-term follow-up was not done. Moreover, other biochemical parameters, other vitamin levels, and association with other comorbidities were not evaluated. In addition, the sample size was relatively small, and the study involved only two centers in the city, which may not represent the whole population.

## Conclusions

In this study, we found that approximately 20% of patients on chronic hemodialysis had vitamin B12 deficiency. Additionally, vitamin B12 deficiency was related to duration of hemodialysis. Consequently, routine serum vitamin B12 level testing in patients with renal failure should be advised, and all treating nephrologists should be aware of the symptoms associated with vitamin B12 deficiency.
